# Effects of glucosamine and risedronate alone or in combination in an experimental rabbit model of osteoarthritis

**DOI:** 10.1186/1746-6148-10-97

**Published:** 2014-04-26

**Authors:** María Permuy, David Guede, Mónica López-Peña, Fernando Muñoz, Antonio González-Cantalapiedra, Jose-Ramón Caeiro

**Affiliations:** 1 Clinical Sciences Department, Veterinary Faculty, University of Santiago de Compostela, 27002 Lugo, Spain; 2 Trabeculae S.L., Ourense, Spain; 3 Cooperative Research Thematic Network in Ageing and Frailty (RETICEF), Carlos III Health Institute, Ministry of Economy and Competitiveness, Madrid, Spain; 4 Orthopaedic Surgery Service, USC University Hospital Complex, Santiago de Compostela, Spain

**Keywords:** Bisphosphonate, Bone, Cartilage, Glucosamine sulfate, Osteoarthritis, Risedronate, Synovial membrane

## Abstract

**Background:**

The osteoarthritis (OA) treatment in humans and in animals is a major orthopaedic challenge because there is not an ideal drug for preserving the joint structure and function. The aim of this study was to assess the effects of the treatment with oral glucosamine and risedronate alone or in combination on articular cartilage, synovial membrane and subchondral bone in an experimental rabbit model of OA. Osteoarthritis was surgically induced on one knee of 32 New Zealand White rabbits using the contralateral as healthy controls. Three weeks later treatments were started and lasted 8 weeks. Animal were divided in four groups of oral treatment: the first group received only saline, the second 21.5 mg/kg/day of glucosamine sulfate, the third 0.07 mg/kg/day of risedronate; and the fourth group both drugs simultaneously at the same dosages. Following sacrifice femurs were removed and osteochondral cylinders and synovial membrane were obtained for its histological and micro-CT evaluation.

**Results:**

Sample analysis revealed that the model induced osteoarthritic changes in operated knees. OA placebo group showed a significant increase in cartilage thickness respect to the control and inflammatory changes in synovial membrane; whereas subchondral bone structure and volumetric bone mineral density remained unchanged. All the treated animals showed an improvement of the cartilage swelling independent of the drug used. Treatment with glucosamine alone seemed to have no effect in the progression of cartilage pathology while risedronate treatment had better results in superficial fibrillation and in resolving the inflammatory changes of the tissues, as well as modifying the orientation of trabecular lattice. The combination of both compounds seemed to have additive effects showing better results than those treated with only one drug.

**Conclusions:**

The results of this animal study suggested that glucosamine sulfate and risedronate treatment alone or in combination may be able to stop cartilage swelling. The risedronate treatment could partially stop the fibrillation and the inflammation of synovial membrane as well as modify the orientation of trabeculae in healthy and in osteoarthritic knees.

## Background

Among the wide range of pharmacological treatments for osteoarthritis (OA), the disease-modifying drugs, also called SYSADOA (Symptomatic Slow Action Drugs for Osteoarthritis), have been shown to relieve the symptoms and progression of OA. The beginning of their action is slow, usually from the sixth week, and their effect continues over a period of time after stopping treatment. Included in this group of drugs, glucosamine has demonstrated its efficacy and clinical relevance in several clinical trials [1-3] and animal models [4,5] helping to restore the proteoglycan matrix of the articular cartilage, to protect damaged cartilage from metabolic impairment [6] and having a mild anti-inflammatory activity [4]. Glucosamine is an endogenous aminomonosaccharide synthesized by chondrocytes from glucose and basic precursor of the structure of glycosaminoglycans and proteoglycans, which form part of the non-cellular connective tissue. This component is primarily responsible for the mechanical function of cartilage. There are several molecular presentations for glucosamine preparations, although the results are more favourable for glucosamine sulfate than for glucosamine hydrochloride [7].

Bisphosphonates (BPs), non-hydrolysable analogues of inorganic pyrophosphate, have been approved for the treatment of pathologies with an increased bone turnover (like osteoporosis or Paget's disease). They inhibit bone resorption by causing the osteoclast to internalize the bisphosphonate and inducing its apoptosis. There are many clinical and experimental evidences of other biological effects of BPs, which may act on other cells of the joint, such as macrophages or chondrocytes. The bisphosphonates seems to have a chondroprotective effect [8,9], to inhibit matrix metalloproteinases [10-12] or even to inhibit cytokines [13,14] and have been reported to exert chondroprotective and analgesic action in OA [15] even though the exact mechanisms remained unclear. Risedronate or risedronic acid is one of the most potent BPs and has demonstrated several beneficial effects on OA progression. It was reported that select combinations of risedronate and non-steroidal antiinflammatory drug therapy in the early stages of OA preserve trabecular bone mass and reduce the impact of osteophyte bony adaptations and bone marrow lesion-like stimulus in a rat model [16]. Risedronate has also shown a reduction of bone mineral loss at sites where the medial cruciate ligament attaches to bone [17] and a conservation effect on periarticular bone and ligament mechanical properties [18] in rabbit models. Studies in guinea pig models suggest smaller cartilage lesions in risedronate-treated joints versus control groups [19]. The efficacy of risedronate in the treatment of human OA was investigated on some clinical trials. BRISK (British Study of Risedronate in Structure and Symptoms of Knee OA) study [20] revealed clear trends towards improvement in both joint structure and symptoms in patients with primary knee OA treated with risedronate, but KOSTAR (Knee OA Structural Arthritis) study [21] shown that risedronate (compared with placebo) did not improve signs or symptoms of OA, nor did it alter its progression, although a reduction in the level of cartilage degradation biochemical markers was observed.

Glucosamine sulfate and sodium risedronate, administered individually or in combination, act directly on the joint structures affected by osteoarthritis (OA). The aim of this study was to determine the magnitude of changes that occur in the knee joint in the early stages of osteoarthritis and evaluate the effects of the glucosamine or risedronate administration on the joint structure in a rabbit model of OA. Furthermore, it was desired to confirm whether the administration of glucosamine in combination with an antiresorptive therapy like risedronate is able to improve the results obtained with the single administration of glucosamine.

## Methods

### Experimental animal model

Thirty-two healthy adult female New Zealand White rabbits (Granja San Bernardo, Navarra, Spain) of 6–7 months of age and mean weight 5 Kg were used in this study after approval of the protocol by the Ethical Committee of the University of Santiago de Compostela (Spain). The rabbits housing, daily monitoring and experimental procedures were conducted in the Animal Experimentation Service Facility of the Santiago de Compostela University (Lugo, Spain) by accredited veterinarians trained in laboratory animal science. All animal handling and experimentation was performed in accordance with Spanish and European Union regulations about care and use of research animals and this paper has been written following the ARRIVE guidelines [22].

After three weeks of quarantine OA was induced by anterior cruciate ligament transection (ACLT) and partial medial meniscectomy on one knee randomly chosen using the contralateral joint as healthy control. To perform the surgical procedure, animals were firstly pre-medicated with a combination of medetomidine (50 μg/Kg IM, Domtor, Esteve, Barcelona, Spain) and ketamine (25 mg/Kg IM, Imalgène 1000, Merial, Toulouse, France) and anesthetized using isoflurane general anaesthesia (Inspiratory Fraction ISO 2.5-4%, Isova-vet, Schering-Plough, Madrid, Spain). Each animal received peri- and post-operative analgesia using buprenorphine (1 mg/Kg IM, Buprex, RB Pharmaceuticals, Berkshire, UK), antibiotic prophylaxis during one week with enrofloxacin (15 mg/Kg SC once a day, Ganadexil 5%, Invesa, Barcelona, Spain) and pain control with meloxicam (20 μg/Kg SC, Metacam, Boehringer Ingelheim España, Barcelona, Spain) during three days. During all the study animals were housed in cages, allowed to perform normal activity and monitored once a day by trained staff to asses changes in general health.

**Figure 1:**
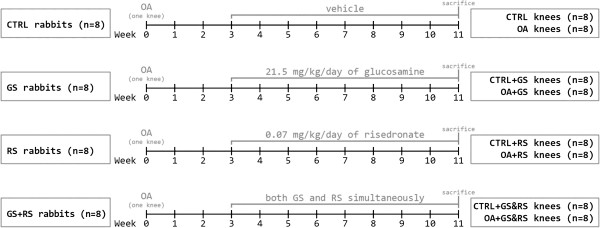
**Experimental design.** Rabbits were divided into four groups, which receive different treatments. Operated joints constituted OA groups, while contralateral joints were used as healthy controls. CTRL: control (healthy); GS: glucosamine sulphate; RS: risedronate; GS&RS: glucosamine sulphate and risedronate applied simultaneously.

Treatments began 3 weeks after surgery. Drugs were orally administered directly into the mouth diluted in saline solution (NaCl) 0.9% during 8 weeks. The animals were randomly divided into 4 experimental groups of 8 animals each: the first group received only vehicle, the second 21.5 mg/kg/day of glucosamine sulfate, the third 0.07 mg/kg/day of risedronate, and the fourth group was treated with both drugs simultaneously (at doses indicated above). Operated joints formed OA groups and contralateral joints healthy groups (Figure 1).

### Necropsy and preparation of histological samples

Rabbits were sacrificed by a sodium pentobarbital overdose (100 mg/Kg IV, Dolethal, Vétoquinol especialidades veterinarias SA, Madrid, Spain) after sedation with ketamine (25 mg/Kg IM, Imalgène 1000, Merial, Toulouse, France). All complete knee joints were removed and immersed in 10% buffered formalin.

After dissecting the articulation, three different samples were obtained from each joint: a synovial membrane section adjacent to the patellar ligament and two 2.9 mm diameter and 8 mm length cores of bone and cartilage from the medial femoral condyle, took at the same anatomical locations in every specimen, using a bone trephine. The first bone cylinder was decalcified (Osteodec, Bio-Optica, Milano, Italy) and together with the articular capsule, were paraffin embedded and sectioned using a microtome (Leica RM 2255, Leica Biosystems Nusshoch GmbH, Germany). Slides were stained with hematoxilin-eosin (H-E) and in addition with safranin O-fast green in the case of the bone and cartilage cylinder.

The second cylinder was initially used to assess the 3D architecture of the cartilage and subchondral bone by micro-computed tomography (micro-CT) and latter processed for undecalcified ground sections in conformity with the method described by Donath [23]. Summarily, the specimens were dehydrated in ascending grades of alcohol, infiltrated and embedded with a light curing resin (Technovit 7200-VLC, Heraus Kulzer GmbH, Werheim, Germany), sectioned and polished using a grinding machine (EXAKT Apparatebau, Norderstedt, Germany) up to approximately 40 μm in thickness and stained using the Lévai-Laczkó method [24].

All the sections were observed using light microscopy and a PC-based image capture system (BX51, DP71, Olympus Corporation, Japan) for histometrical analysis.

### Microscopic evaluation

#### Undecalcified sections

**Figure 2:**
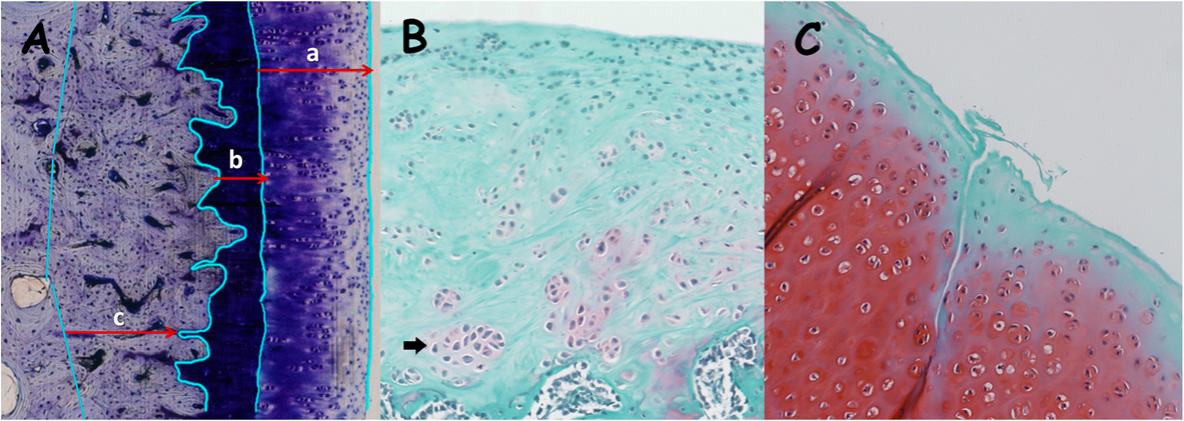
**Microscopic evaluation. 2A** (undecalcified sections): measurement of cartilage and subchondral bone cortical thickness (a: nCg.Th; b: cCg.Th; c: SB.Th). **2B** and **2C** (decalcified sections): Assessment of chondrocyte clusters (black arrow in Figure 2B), grade of safranin stain in samples (**2B** and **2C**), alterations in cartilage surface (**2C**).

Quantitative histology was performed using undecalcified sections applying morphometrical parameters previously published [25], by a masked examiner and using PC-based image analysis programs (Cell-sens 1,5 (Olympus Corporation, Japan) and Micro-image 4.0 (Media cybernetics, Bethesda, MD, USA). The parameters evaluated were:

(A) Subchondral cortical bone thickness (SB.Th) and cartilage thickness (Cg.Th). SB.Th is defined as the mean distance between the cartilage and the subchondral cortical bone limits. Cartilage thickness was divided in non-calcified cartilage thickness (nCg.Th) and calcified cartilage thickness (cCg.Th) using the tidemark as reference. All were calculated as mean distances (Figure 2A).

(B) Surface undulations of the cartilage (FI), defined as the difference in between the length of the upper margin of the cartilage and the width of the same measurement area.

(C) Trabecular subchondral bone area (Tb.A) and trabecular separation (Tb.Sp) in subchondral bone. To obtain these measurements, a region of interest (ROI) was defined as a 4 × 2 mm area beginning immediately after the subchondral cortical bone. Tb.A was defined as the percentage of trabecular bone in this region and the Tb.Sp the mean distance between trabeculae measured on the diagonal of the ROI (Tb.Sp = (1/Tb.N)-Tb.Th).

#### Decalcified sections

Synovial membrane and decalcified sections of bone core biopsies, including cartilage, were used for evaluating articular changes. The evaluation was always performed by two independent observers following guidelines already published [26,27]. The parameters evaluated were:

(A) Severity of cartilage pathology: Grade of alteration of cartilage surface (Figure 2C).

(B) Severity of chondrocyte pathology: Number and distribution of chondrocytes in cartilage (Figure 2B).

(C) Severity of proteoglycan pathology: depth of the “red” colour of the staining (Figure 2B and Figure 2C).

(D) Tidemark integrity.

(E) Microscopic changes of synovial membrane: evaluating the lining cells, presence of hyperplasia and cellular inflammatory infiltration.

The gradation of the affectation of structures in these decalcified samples was as follows: in cartilage, chondrocyte and proteoglycan pathology from 0 (normal) to 4 (completely affected) and in tidemark and synovial membrane from 0 to 2.

### Micro-computed tomography

Subchondral bone cores containing articular cartilage were assessed with high-resolution X-ray micro-CT (SkyScan 1172, Bruker micro CT NV, Kontich, Belgium) in Trabeculae S.L. research lab (Ourense, Spain). The X-ray source was set at 50 kV and 200 μA, with a pixel size of 12 μm and the use of a 0.5 mm aluminium filter. Images were reconstructed based on Feldkamp algorithm [28], and segmented into binary images using adaptive local thresholding methods. Standard indices of cancellous bone microstructure were determined [29], including bone volumetric fraction (BV/TV), trabecular thickness (Tb.Th) and separation (Tb.Sp), trabecular number (Tb.N), trabecular bone pattern factor (Tb.Pf), structural model index (SMI), and degree of anisotropy (DA).

Volumetric bone mineral density (vBMD) was determined in the analysed region of subchondral bone by calibration against hydroxyapatite phantoms of known density. Two phantoms, 250 and 750 mg/cm^3^ of hydroxyapatite, were scanned under the same conditions than bone samples. An estimation of vBMD of each of the samples was obtained comparing the attenuation coefficients of the phantoms and bone.

An additional scan was made for each sample with in order to correctly visualize the non-calcified part of the articular cartilage and measuring its volume (nCg.V) and mean thickness (nCg.Th) three-dimensionally. For this scans, X-ray source was set at 60 kV and 167 μA, voxel size was reduced until 5 μm, and no filter was used.

### Statistical approach

Results were expressed as mean ± standard deviation. The normality of the data was assessed using the Shapiro-Wilk test. Levene's test was used to assess the equality of variances of normal variables and the statistical comparison was performed using ANOVA. Post-hoc analysis was made by Tukey's HSD test or by Games-Howell test for parameters with equal or different variances respectively. For non-normal variables, statistical comparison was performed using Kruskal-Wallis H test and post-hoc analysis using Dunn’s test. All statistical analyses were performed using commercially available software IBM SPSS Statistics 19 (IBM, Armonk, NY, USA). Differences were considered significant when *p* < 0.05.

## Results

During the procedure no changes in weight or general condition were observed. All animals tolerated well the treatments.

From a total of 64 joints, one had to be excluded due to an infection (OA group); the rest 63 presented an adequate status to be histologically analysed so no modification of the original protocol was necessary.

### Histology quantitative results

**Figure 3:**
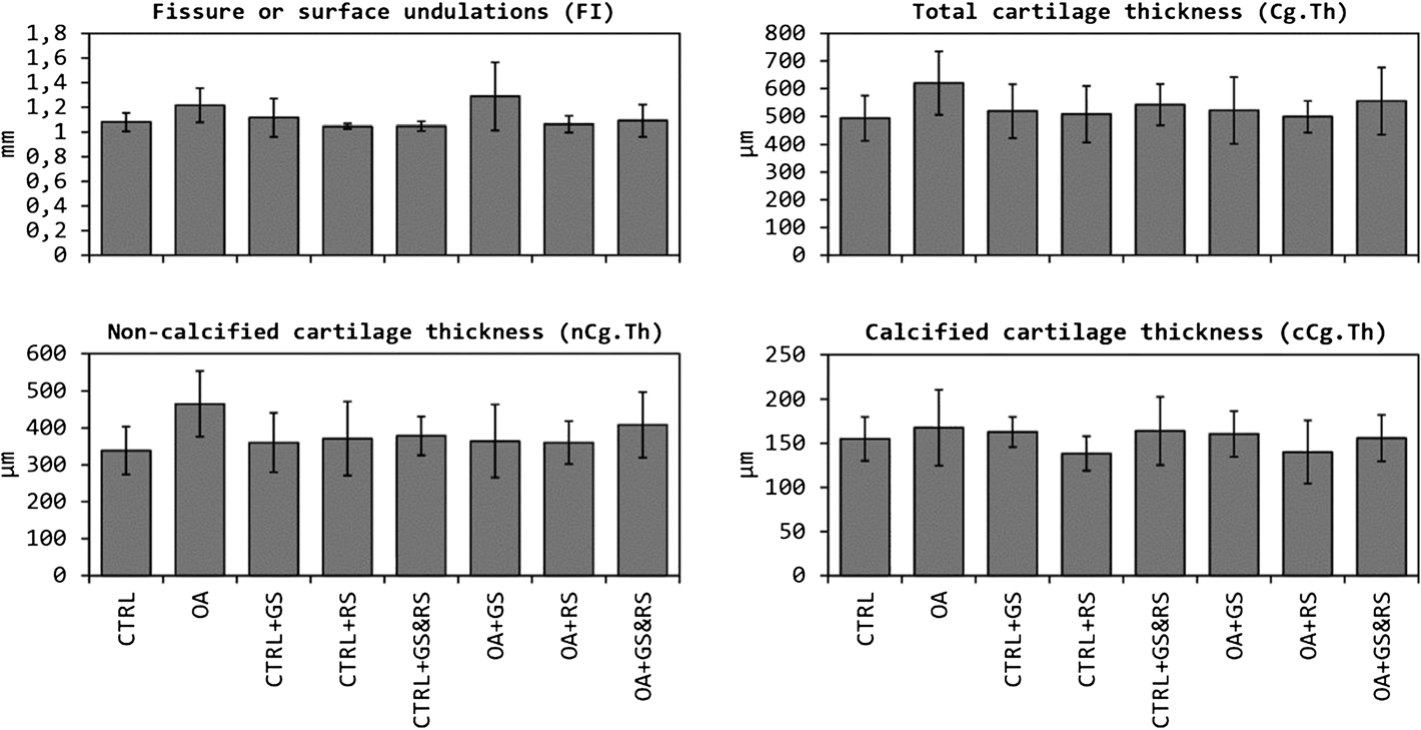
**Comparison between the histological parameters of the cartilage obtained from undecalcified samples.** CTRL: healthy + placebo; OA: osteoarthritis + placebo; CTRL + GS: healthy + glucosamine; OA + GS: osteoarthritis + glucosamine; CTRL + RS: healthy + risedronate; OA + RS: osteoarthritis + risedronate; CTRL + GS&RS: healthy + combined treatment of glucosamine and risedronate; OA + GS&RS: osteoarthritis + combined treatment of glucosamine and risedronate.

**Figure 4:**
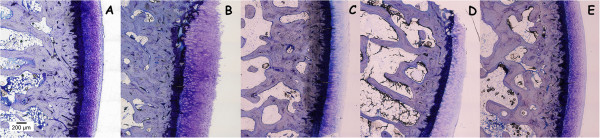
**Representative histology images of Lévai**-**Laczkó stained undecalcified sections.** Magnification 10x. **A**: CTRL; **B**: OA; **C**: OA + GS; **D**: OA + RS; **E**: OA + GS&RS. Observe the reduction in cartilage thickness in the treatment groups **(C, D, E)** in regard to the OA group **(B)**.

No parameters measured in cartilage (Figure 3) and in subchondral bone (data not shown) showed significant differences; although appears to be a little tendency to thickening of the cartilage in the OA group respect to controls standing the results of the three OA treatment groups (OA + GS, OA + RS, OA + GS&RS) in between OA and normal (Figure 4). Respect to surface undulations (FI) there were no statistical differences between groups but OA and OA + GS have more surface fibrillation than controls, OA + RS and OA + GS&RS.

Although the results discussed above were not statistically significant, in the microscopic images of the calcified samples was possible to observe several differences between groups (Figure 4). The thickness of the cartilage in the OA group (4B) was greater than in the three treatment groups (4C, 4D, 4E) being these groups more similar to the control (4A). The distribution of the chondrocytes in the cartilage was different too, showing more disorganization in the OA group (4B) than in the others. Finally in the OA picture we could see the loss of the part of the calcified cartilage as well as in the glucosamine treated group (4C) and not in the others.

### Histology qualitative results (Table 1)

**Table 1:** **Comparison between experimental groups of the different scores for microscopic grading of cartilage and synovial alterations**, **obtained from decalcified samples**

**CONTROL**	**CTRL**	**CTRL** **+** **GS**	**CTRL** **+** **RS**	**CTRL** **+** **GS&RS**
	Median (25, 75)	Median (25, 75)	Median (25, 75)	Median (25, 75)
**Microscopic grading of cartilage alterations**				
Severity of cartilage pathology	**0 (0, 0.5)**^**e,f,g**^	**0 (0, 0)**^**c,e,f,g**^	**0 (0, 0.5)**^**b,e,f,g**^	**0 (0, 1)**^**e,f,g**^
Severity of chondrocyte pathology	0 (0, 1)	0.5 (0, 1.25)	0 (0, 1)	0.5 (0, 1)
Severity of proteoglycan pathology	0 (0, 0)	0.5 (0, 1)	0 (0, 0)	0 (0, 0)
Tidemark integrity	1 (0.75, 2)	0.5 (0, 1)	1 (0, 1)	1 (1, 1)
**Microscopic grading of synovial changes**				
Lining cells characteristics	**0 (0, 0)**^**e,f,h**^	**0 (0, 0)**^**e,f,h**^	**0 (0, 0)**^**e,f,h**^	**0 (0, 0)**^**e,f,h**^
Presence of hyperplasia	**0 (0, 0)**^**e,f,g,h**^	**0 (0, 1)**^**e,f,h**^	**0 (0, 0)**^**e,f,g,h**^	**0 (0, 0)**^**e,f,g,h**^
Cell infiltration characteristics	**0 (0, 0)**^**e,f,g**^	**0 (0, 0)**^**e,g**^	**0 (0, 0)**^**e,f**^	**0 (0, 0)**^**e,f**^
				
**OSTEOARTHRITIS**	**OA**	**OA** **+** **GS**	**OA** **+** **RS**	**OA** **+** **GS&RS**
	median (25, 75)	median (25, 75)	median (25, 75)	median (25, 75)
**Microscopic grading of cartilage alterations**				
Severity of cartilage pathology	**1 (0.5, 1.5)**^**a,b,c,d,h**^	**1 (1, 2)**^**a,b,c,d,h**^	**1 (0.5, 1.5)**^**a,b,c,d,h**^	**0 (0, 0.625)**^**e,f,g**^
Severity of chondrocyte pathology	1 (0.5, 1.75)	1 (1, 3)	1 (0.5, 1.5)	1 (0, 1.125)
Severity of proteoglycan pathology	1 (0, 2)	1 (0.5, 3)	1 (0, 2.75)	3 (0.75, 4)
Tidemark integrity	0 (0, 1.5)	0 (0, 0)	1 (1, 1)	1 (0.25, 1)
**Microscopic grading of synovial changes**				
Lining cells characteristics	**1 (1, 1)**^**a,b,c,d,g,h**^	**1 (0, 2)**^**a,b,c,d,g,h**^	**0 (0, 0.25)**^**e,f**^	**0 (0, 0.625)**^**a,b,c,d,e,f**^
Presence of hyperplasia	**1 (1, 1.75)**^**a,b,c,d,f,g,h**^	**1.5 (1, 2)**^**a,b,c,d,e,g,h**^	**0.5 (0, 1)**^**a,c,d,e,f**^	**1 (0, 1)**^**a,b,c,d,e,f**^
Cell infiltration characteristics	**1 (0.25, 1)**^**a,b,c,d,h**^	**0.75(0, 1)**^**a,b,c,d,g,h**^	**0 (0, 0.25)**^**a,f**^	**0 (0, 0.25)**^**e,f**^

CTRL: healthy + placebo; OA: osteoarthritis + placebo; CTRL + GS: healthy + glucosamine; OA + GS: osteoarthritis + glucosamine; CTRL + RS: healthy + risedronate; OA + RS: osteoarthritis + risedronate; CTRL + GS&RS: healthy + combined treatment of glucosamine and risedronate; OA + GS&RS: osteoarthritis + combined treatment of glucosamine and risedronate. Statistical differences p < 0.05: ^a^ vs. CTRL, ^b^ vs. CTRL + GS, ^c^ vs. CTRL + RS, ^d^ vs. CTRL + GSRS, ^e^ vs. OA, ^f^ vs. OA + GS, ^g^ vs. OA + RS, ^h^ vs. OA + GSRS.

**Figure 5:**
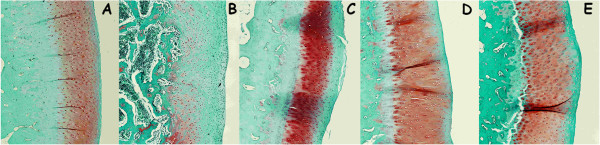
**Representative histology images of safranin O stained sections.** Magnification 20x. **A**: CTRL; **B**: OA; **C**: OA + GS; **D**: OA + RS; **E**: OA + GS&RS. Observ the better conservation of the normal characteristics of the cartilage in the two risedronate treated groups **(D, E)** respect to OA **(B)** and GS **(C)**.

In cartilage pathology there was statistical significance between control groups and three of the treatment groups (OA, OA + GS and OA + RS) but not with OA + GS&RS. This group (OA + GS&RS) had not differences with any control group but it presented differences with the other OA groups. The severity of chondrocyte pathology, the proteoglycan pathology and the tidemark integrity, showed no statistical changes between groups (Figure 5); but, although the differences were not significant, in the OA group there is a loss of the proteoglycan content in all the cartilage (as show in Figure 5B) which is less pronounced in the treatment groups, as well as a better conservation of the normal distribution of the chondrocytes in rows in the treatment groups (5C, 5D, 5E).

**Figure 6:**
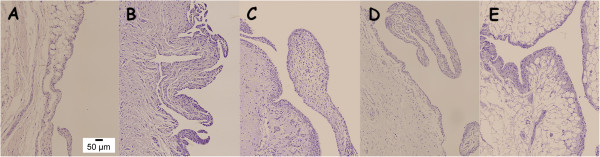
**Representative histology images of synovial membrane sections.** Magnification 20x. **A**: CTRL; **B**: OA; **C**: OA + GS; **D**: OA + RS; **E**: OA + GS&RS. In the risedronate treated groups **(D and E)** the characteristics are more similar to control **(A)**, in glucosamine treated **(C)** were present infiltrations of inflammatory cells as in OA **(B)**.

The three variables measured in synovial membrane (Figure 6) showed significant differences between CTRL and OA placing the treatment groups in between them with different results. In lining cell characteristics risedronate treated osteoarthritic groups (OA + RS and OA + GS&RS) had differences with OA group, but not glucosamine osteoarthritic group (OA + GS). The OA + RS showed not differences with any control group and the OA + GS&RS group had differences with all the groups (healthy and OA) unless with OA + RS.

In hyperplasia the CTRL group had differences with the four OA groups and the OA placebo group with all the rest (controls and osteoarthritic), standing the results of the three treatment groups in between OA and healthy. The glucosamine OA group (OA + GS) had differences between the two risedronate treated groups (OA + RS and OA + GS&RS) but these two did not differ between them.

With respect to the cell infiltration in synovial membrane, the CTRL placebo group showed differences with OA, OA + GS and OA + RS but not with OA + GS&RS and this one showed differences with OA group and with OA + GS standing closer to normality than the other surgically induced groups.

### Micro-CT results

**Figure 7:**
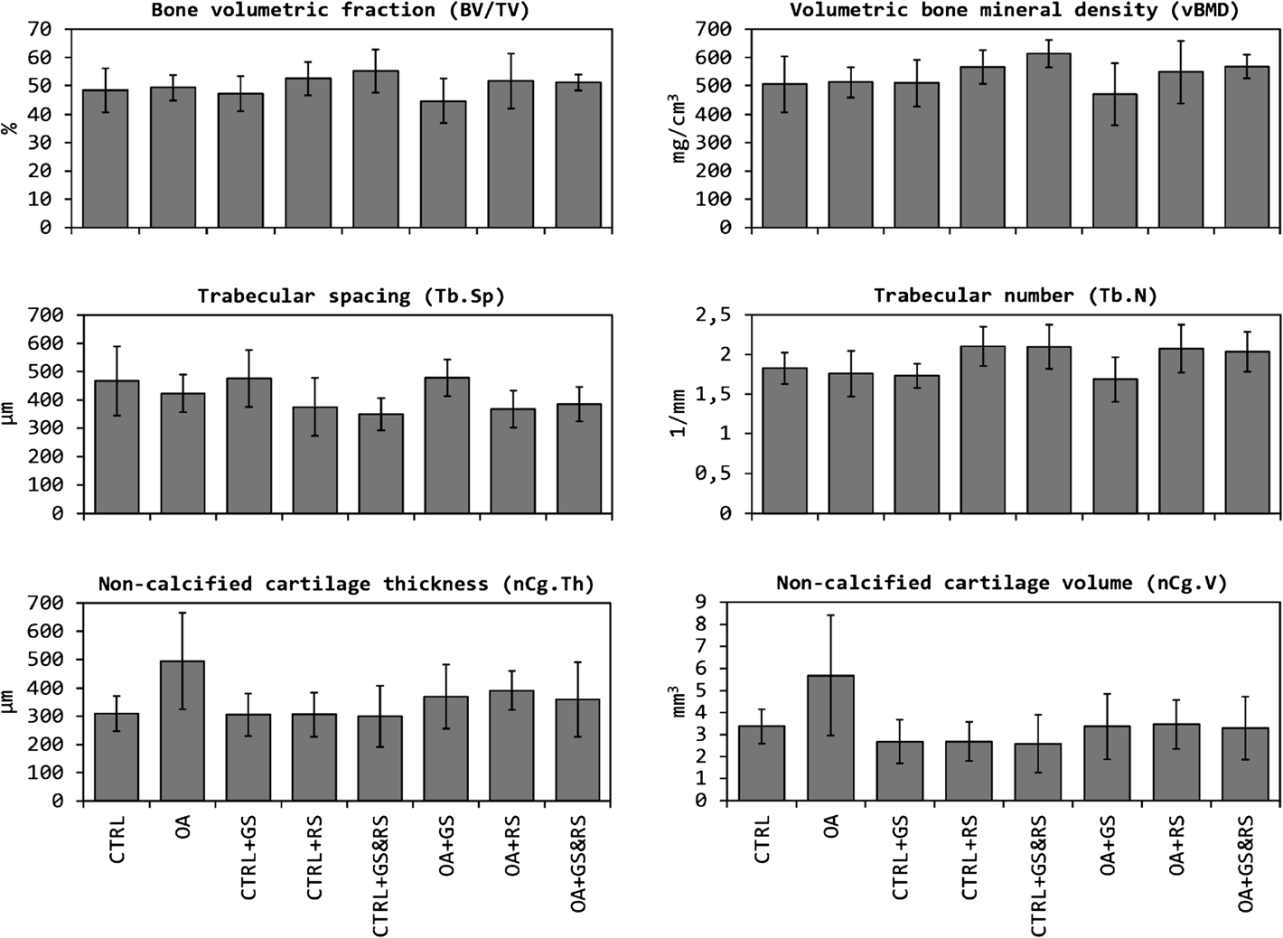
**Comparison between the micro**-**CT parameters.** CTRL: healthy + placebo; OA: osteoarthritis + placebo; CTRL + GS: healthy + glucosamine; OA + GS: osteoarthritis + glucosamine; CTRL + RS: healthy + risedronate; OA + RS: osteoarthritis + risedronate; CTRL + GS&RS: healthy + combined treatment of glucosamine and risedronate; OA + GS&RS: osteoarthritis + combined treatment of glucosamine and risedronate.

Micro-CT data is shown on Figure 7. The results obtained in the osteoarthritic group (OA) compared to the healthy control group (CTRL) showed that there were no differences in any of the microstructural parameters referred to subchondral bone, or in volumetric bone mineral density (vBMD). However, non-calcified cartilage thickness (nCg.Th) was significantly increased in the OA group (*p* < 0.05).

In healthy samples treated with glucosamine (CTRL + GS) has not been observed any change in the structural properties of the subchondral bone compared to CTRL group. The morphology of the cartilage did not differ between both groups. Treatment with GS in osteoarthritic joints (OA + GS) caused no significant changes in subchondral bone versus untreated osteoarthritic group (OA), however it reduced the thickness of cartilage compared to OA group (*p* < 0.05) reaching intermediate values between CTRL and OA groups, i.e. showing significant differences versus CTRL group too (*p* < 0.05).

Healthy group treated with risedronate (CTRL + RS) compared with the CTRL group, presented a reduction of the degree of anisotropy (*p* = 0.000). Both BV/TV and vBMD were increased in the treated group, but the differences compared to CTRL group did not become significant. Cartilage structure was neither altered in the CTRL + RS group. By the other hand, in OA samples, risedronate appeared to have similar effects on the subchondral bone. The only parameter which revealed significant differences between OA and OA + RS groups was the degree of anisotropy (*p* = 0.001). These data suggested that treatment with risedronate alter trabeculae orientation, becoming trabecular lattice more disorganized than in untreated groups, diminishing trabecular separation and increasing trabecular number. But surprisingly, we have observed changes in the morphology of the cartilage. nCg.Th in OA + RS group was smaller than that of OA group (*p* < 0.05), although showing differences also versus CTRL group (*p* < 0.05).

In the healthy group treated with the combination of both drugs (CTRL + GS&RS) the only change in subchondral bone was on DA (*p* = 0.000 versus CTRL), probably due to the effect of risedronate described above. BV/TV and vBMD were again increased in the treated group versus CTRL group but they did not show significant differences. The combination of both drugs did not alter the structure of the cartilage. In OA samples, the effect of combined therapy on bone was again a decrease in DA (*p* = 0.000 versus OA). In relation to the cartilage, combined therapy was also able to stop swelling in OA + GS&RS group, reducing nCg.Th value, which showed significant differences versus OA and CTRL groups (*p* < 0.05 in both cases).

## Discussion

Different animal models have been widely used for the study of the efficacy of therapies to improve, resolve or prevent the OA. The surgically induced models (joint instability) produce a gradual progression of the degenerative changes in the joint that mimic the pathogenesis and pathology of the human traumatic OA [30,31]. The rabbit model was extensively used for testing potential chondroprotective agents including bisphosphonates [18,32] and glucosamine [4,33]. Previously published data showed that most rabbits with anterior cruciate ligament transection develop cartilage degeneration [33,34] and subchondral bone alterations [33] as soon as 8 weeks postsurgery. In the present study, the development of OA was not still advanced at eleven weeks postsurgery, although it was enough to produce detectable changes in the articular cartilage but not in the subchondral bone. Articular cartilage appears in a state of swelling -characteristic of the early stages of the disease- previous to its erosion and destruction [35]. The subchondral bone, however, had not the typical sclerosis associated with this pathology, not appearing any differences between the healthy control group and the untreated OA group in any of the microstructural parameters analysed by either histology or micro-CT.

In animal models of OA histological assessment of articular cartilage, using mainly Mankin [36] or modified Mankin scoring systems in decalcified samples, was considered the gold standard for the evaluation of the presence, extent and severity of the pathology [25]. However, because this is a subjective scoring system with the of inter- and/or intra-observer variability, the histomorphometry of calcified samples, using computer analysis systems, was introduced with a greater degree of objectivity and reproducibility [25]. Micro-CT had become in the last years the gold standard for the three-dimensional analysis of bone microstructure but with soft tissues as the articular cartilage the technique presents problems due to its low x-ray transmission. Normally to quantify the structure of cartilage using micro-CT, was necessary the use of complex staining methods with radiopaque contrast agents [37-39]. For the present study we used quantitative (histomorphometry) and qualitative microscopic evaluations and also micro-CT scanning, to analyse the structure of subchondral bone and articular cartilage in calcified and non-calcified samples in all the groups of treatment. For the evaluation with micro-CT we had adjusted the scan conditions for a visualization of articular cartilage enough to be able to quantify its morphology without the use of contrast techniques. Mineralized tissue was usually scanned at 50 KV and 200 μA of the X-ray source, and with the use of aluminium filters. After a series of trials to get the visualization of cartilage, we decided to increase the voltage to 60 KV, decrease the intensity to 167 μA, and remove the aluminium filter. In this way we have scanned the articular cartilage obtaining valid images for a proper binarization and to quantify its thickness and volume. Due to different scanning conditions required to visualize subchondral bone and cartilage, each sample was scanned twice (once for each of the conditions mentioned above). This is the first time, to the best knowledge of the authors, that the morphology of articular cartilage is quantified by micro-CT without the use of contrast agents.

The efficacy of the glucosamine treatment for OA was tested in several *in vitro* and *in vivo* models as well as in clinical trials. *In vitro*, Bassler *et al*. [40] showed a stimulatory effect of glucosamine on the biosynthetic activity on human chondrocytes probably due to the inhibition of the degradation of proteoglycans and the stimulation of its synthesis [41,42]. This biosynthetic activity seen *in vitro* was not always corresponded with the findings in animal models and in clinical trials in which some supported the hypothesis that glucosamine is a symptom-modifying agent for OA [43,44] while others had detected no effect [45,46]. In our study the glucosamine sulfate treatment alone seemed not alter the structure of subchondral bone measured by histomorphometry and by micro-CT nor in healthy samples nor in OA ones. Even if glucosamine sulfate treatment did not seem to have any structural effect on cartilage healthy samples, in OA was able to partially stop the swelling of articular cartilage reaching intermediate values of cartilage thickness (Cg.Th) between CTRL and OA groups (measured in calcified samples) although the results are not statistically significant. Using histomorphometry we could observe that in OA + GS samples the surface undulations (FI) were more elevated than in other treatment groups, but still lower than the OA group; this findings were in conformity with those of other groups who reported that the administration of glucosamine did not prevent fibrillation and/or erosions of the articular cartilage in an ACLT model in rabbits although there was an overall trend toward a reduction in the severity of the disease [2,5]. With respect to the severity of changes in synovial membrane, which represents the anti-inflammatory effect of the drug, we could see that the values of the OA + GS group were similar to the OA group (both had statistical differences with the control groups) so in our case, the drug did not show the expected anti-inflammatory action in the synovial membrane (This results differ from those of Pavelka *et al*. [2] where they observed improvement of synovitis).

Early in the pathogenesis of OA a period of periarticular osteopenia was developed prior to the latter stage of subchondral bone sclerosis [47]; this osteopenia had also been reported after anterior cruciate ligament injury in clinical trials [48] and a significant reduction in the bone mineral density was reported in patients with mild OA when it was compared with healthy ones [49]. With the purpose of inhibiting bone remodelling and consequent osteopenia, bisphosphonates were proposed as a possible treatment for OA in the early stages of the disease because they may help in preserving periarticular bone mechanical properties [50]. Risedronate, a potent aminobisphosphonate, was shown its effectiveness in conserving periarticular bone properties in animal models of OA [50]. In the present study in rabbits, risedronate administered alone or in combination with glucosamine seemed to considerably modify the orientation of trabecular lattice both in healthy and OA subchondral bone, measured by micro-CT (groups CTRL + RS, CTRL + GS&RS, OA + RS and OA + GS&RS), represented as a diminution in Tb.Sp and an increase in Tb.N showing a trend to bone formation in animals treated with risedronate, as was previously published by other authors [50,51]. When the cartilage was assessed, in calcified samples measured by micro-CT and quantitative histomorphometry, risedronate was able, at least in part, to reduce the cartilage swelling perhaps due to an anti-inflammatory activity [14] reaching intermediate values between control and OA, and, as seen in histomorphometry the values of superficial fibrillation were similar to those in the controls and lower than in the OA ones. In the safranin-O/fast green stained samples, the results for the groups treated with risedronate were in between OA and controls suggesting an improvement of the cartilage pathology. Similar results as those obtained in the present study were reported previously; Myers *et al*. [52] established that the nitrogen-containing bisphosphonates reduced early turnover of cancellous and subchondral bone in the canine anterior cruciate ligament transection model but in this model mild OA changes in cartilage were detectable 12 weeks post-surgery. In the present study we found a favourable effect of risedronate treatment (alone and in combination with glucosamine) on the inflammatory changes in the synovial membrane. The anti-inflammatory and pain relieving efficacy of bisphosphonates was reported before [53] but these studies revealed an anti-inflammatory activity not related to ameliorating the synovitis [54].

## Conclusions

Oral risedronate and glucosamine sulfate, alone or in combination, were able to improve cartilage swelling in the early stages of OA in a rabbit instability model. Oral risedronate but not glucosamine could preserve cartilage from superficial fibrillation and improve the inflammatory changes in synovial membrane. On subchondral bone, risedronate could modify the orientation of trabecular lattice in all treatment groups (healthy and OA) indicating its effect in bone although it has been administered orally to the rabbits.

The study also demonstrated the validity of the animal model, and that micro-CT could be a valid technique to detect morphological changes in articular cartilage with comparable results to those of histomorphometry.

## Abbreviations

ACLT: Anterior cruciate ligament transection; ARRIVE: Animal research reporting in vivo experiments; BPs: Bisphosphonates; BRISK: British study of risedronate in structure and symptoms of knee osteoarthritis; CT: Computed tomography; KOSTAR: The knee osteoarthritis structural arthritis; OA: Osteoarthritis; ROI: Region of interest; SYSADOA: Symptomatic slow action drug for osteoarthritis.

## Competing interest

The authors declare that they have no competing interests.

## Authors‘ contributions

DG, JRC, FM and AGC participated in the conception and design of the study. The animal model and the histological analyses were performed by MP, ML, FM and AGC; while micro-CT assessments were made by DG and JRC. MP and DG contributed equally to this work. All authors have collaborated on data analysis, interpretation of results, drafting and revising of article and final approval.
